# Enhanced efficiency in hollow core electrospun nanofiber-based organic solar cells

**DOI:** 10.1038/s41598-021-00580-4

**Published:** 2021-10-27

**Authors:** Mohammad Ali Haghighat Bayan, Faramarz Afshar Taromi, Massimiliano Lanzi, Filippo Pierini

**Affiliations:** 1 Department of Biosystems and Soft Matter, Institute of Fundamental Technological Research, Polish Academy of Sciences, 02-106 Warsaw, Poland; 2 Department of Polymer Engineering, Amirkabir University of Technology, 15875-4414 Tehran, Iran; 3 Department of Industrial Chemistry “Toso Montanari”, Alma Mater Studiorum – University of Bologna, 40136 Bologna, Italy

**Keywords:** Nanoscience and technology, Nanoscale devices, Electronic devices

## Abstract

Over the last decade, nanotechnology and nanomaterials have attracted enormous interest due to the rising number of their applications in solar cells. A fascinating strategy to increase the efficiency of organic solar cells is the use of tailor-designed buffer layers to improve the charge transport process. High-efficiency bulk heterojunction (BHJ) solar cells have been obtained by introducing hollow core polyaniline (PANI) nanofibers as a buffer layer. An improved power conversion efficiency in polymer solar cells (PSCs) was demonstrated through the incorporation of electrospun hollow core PANI nanofibers positioned between the active layer and the electrode. PANI hollow nanofibers improved buffer layer structural properties, enhanced optical absorption, and induced a more balanced charge transfer process. Solar cell photovoltaic parameters also showed higher open-circuit voltage (+ 40.3%) and higher power conversion efficiency (+ 48.5%) than conventional architecture BHJ solar cells. Furthermore, the photovoltaic cell developed achieved the highest reported efficiency value ever reached for an electrospun fiber-based solar cell (PCE = 6.85%). Our results indicated that PANI hollow core nanostructures may be considered an effective material for high-performance PSCs and potentially applicable to other fields, such as fuel cells and sensors.

## Introduction

Today, due to rising energy consumption and limited fossil fuels, the need and desire for cost-effective renewable energy has created much interest in the development of new clean energy sources. Renewable energies such as wind turbines, tidal energy, and solar cells are unlimited sources^1^. Photovoltaics (PV) are devices serving to convert solar light into electrical energy using semiconductor materials, which are receiving a great deal of attention today. The advantages of using PV include avoiding the emission of greenhouse gases and toxic substances, as well as little maintenance needed during operation^2^.

Currently, inorganic semiconductors dominate PV technology. The production process of traditional mineral solar cells requires high heat, with a consequently exorbitant production cost. On the other hand, organic solar cells, which are developed using small molecules and conjugated polymers, offer a cost-effective way to convert solar energy into electrical energy^3^. Furthermore, the properties of conjugated molecules and polymers can be controlled by modifying their chemical composition and improving their desired properties. Nevertheless, organic solar cell applications are limited by the low photoconversion efficiency of these devices^4^.

To improve organic solar cell efficiency, various procedures have been implemented in the past few decades. A representative example is the deployment of new organic molecules with an enhanced band gap in their active layer. Other methods, such as post-treatment doping, incorporation of ultrathin semiconductors, and fabrication of tandem solar cells with efficient interlayers, have been developed^5^. Most importantly, light-trapping materials—to enhance light absorption—have also been used and alternative interlayers added^6^. The inclusion of an additional hole transport layer—such as poly(3,4-ethylenedioxythiophene) polystyrene sulfonate (PEDOT:PSS)—is a perfect example^7^. Indeed, the use of a buffer layer has been seen as an effective strategy to improve contact properties and charge activity and to minimize any undesired charge recombination^8^.

As mentioned earlier, of all these polymers, PEDOT: PSS is the most popular polymer due to its good solubility and high conductivity. PEDOT:PSS is actually a polymer blend of two ionomers, where PEDOT is conductive and PSS helps to dissolve in aqueous media^9^. There is a strong coulombic attraction between the two ionomers that causes the mixture to entangle in a coiled state, surrounded by conductive PEDOT oligomers covered by the insulating PSS polymer^10^. Typically, a thin layer of this compound is deposited directly on the surface of the ITO electrode as a hole transport layer^11^.

PANI is one of the most attractive conductive polymers and due to its unique properties, such as optical and magnetic properties, simple polymerization process, chemical, thermal and environmental stability, high electrical conductivity, affordable synthesis and high mobility of charge carriers^12^. The functionality of PANI can be different by doping with different acids and has many applications. The electrical conductivity of the polymer also varies according to the degree of oxidation by doping and protonation^13^. PANI has different structures which have different physical and chemical properties. Due to the oxidative state of the monomer, PANI exists in three different forms: Leucoemeraldine (fully reduced), Emeraldine and Pernigraniline (fully oxidized). Meanwhile, only the emeraldine salt structure is electrically conductive^11^. Imine positions in the open form of emeraldine are easily protonated by doping. This trait leads to a significant increase in conductivity and the formation of positive charges in the polymer network^13^. This, while the number of electrons in the polymer structure remains constant. Thus, protonated emeraldine PANI, the so-called emeraldine salts, give rise to new optical, electrical and magnetic properties^14^. PANI is synthesized by chemical, electrochemical and colloidal methods. However, electrochemical methods such as chemical synthesis are suitable method for the preparation of polymers with a specific structure. The chemical method is preferred to the electrochemical method due to the simplicity and reproducibility of the synthesis process^15^. PANI nanostructures have also been investigated, and the experimental results described by Simotwo et al. show that PANI nanotubes with a small diameter are a very attractive material for use as counter electrodes^16^.

Different morphologies of PANI have been achieved by controlling the synthesis and fabrication conditions^17^. Electrospun fibers have been used in several application fields such as in biomedicals^18^, filtration^19^, catalysis^20^, batteries^21^, and solar cells^22^, due to their nanoscale and/or microscale fiber diameters and highly specific surface area. Electrospinning is a simple, convenient, and inexpensive technique used to prepare PAN and doped PANI nanofibers. Recent advances in electrospinning techniques have made possible the creation of electrospun nanofibers with controllable morphologies such as core/shell, hollow, and porous structures^23^. Long and continuous nanofibers with a uniform diameter and smooth surface have been made available thanks to this method, which has already been successfully applied by our group in the fabrication of high-performance solar cells. However, the production of pure PANI nanofibers is still a challenge, due to the poor spinnability of its solutions. PANI blended with a more spinnable polymer was extensively used to achieve PANI-based nanofibers. The presence of a second non-conductive polymer significantly reduces the final electrical and optical properties of nanomaterials^24^. On the other hand, a sacrificial template offers a fascinating way to fabricate pure conductive polymer nanofibers with a sophisticated architecture by electrospinning^25^.

Compared to other one-dimensional monolithic nanostructures (e.g., nanorods and nanowires), hollow nanofibers have a larger surface area, which increases the number of active centers^26^. Electrospinning technique allows fabricating conductive hollow nanofibers that show outstanding electrical properties due to the higher surface area and the number of free electrons^27^.

In the current study, a new class of hole transport layers (HTLs) was fabricated based on electrospun hollow core PANI nanofibers with high conductivity and solubility, and their possible application was thoroughly investigated. The effect of PANI morphology on polymer energy levels and optical properties was analyzed by cyclic voltammetry (CV) and UV–Vis spectroscopy (UV–Vis). The morphology of the prepared buffer layers for organic solar cells (OSCs) was examined by atomic force microscopy (AFM), and the final device photovoltaic tests showed that hollow PANI decreases the hole extraction route and improves hole-collection efficiency. These findings proved that the new HTL based on hollow core PANI nanofibers is a promising material for charge transport layers in optoelectronic devices, since the open-circuit voltage (Voc) increased by 40%, while its efficiency rose up to 6.85%, which is—to the best of our knowledge—the highest obtained value for an electrospun nanofiber-based organic solar cell.

## Materials and methods

### Materials

All materials and reagents were used as purchased and without any further purification unless otherwise stated. Ethanol (EtOH, 99.5%, Merck), N,N-dimethylformamide (DMF, 99%, Merck), acetone (99%, Merck), hydrochloric acid (HCl, 37%, Merck), deionized water (DI, 99%, Merck), 1,2-dichloroethane (DCE, 99%, Merck), N-methyl-2-pyrrolidone (NMP, 99%, Aldrich), and chlorosulfonic acid (ChA, 99%, Merck) were used during polymer synthesis and fiber fabrication. Aniline (ANI, 99.5%, Aldrich), was purified before use, while ammonium persulfate (APS, 98%, Merck), polyacrylonitrile (PAN, 150,000, Aldrich), PEDOT:PSS (Aldrich), poly(3-hexylthiophene) (P3HT, 97.6%, Aldrich), [6-6]-phenyl-C61-butyric acid methyl ester (PCBM, 99.5%, Aldrich), and glass-coated with ITO (surface resistivity of 40 ± 2 Ω/square) were used as purchased.

### Synthesis and characterization of PANI and sulfonated PANI

**Figure 1:**
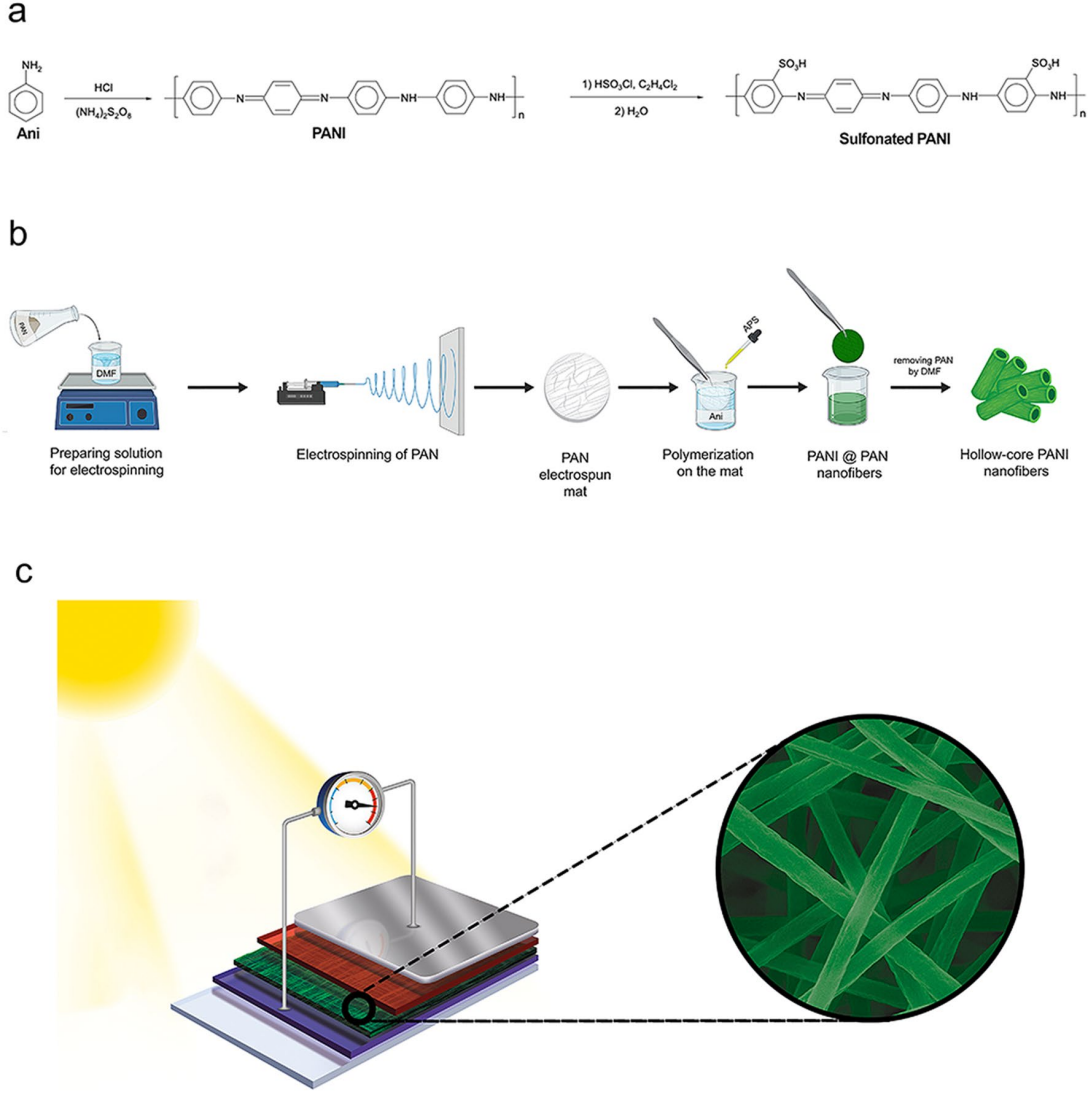
Development of the electrospun nanofiber-based organic solar cell. (**a**) Schematics of the PANI and sulfonated PANI syntheses. (**b**) Graphical representation of the preparation procedures of hollow core PANI nanofibers, combining electrospinning and a facile sacrificial template method. (**c**) Sketch representing the architecture of the organic solar cell. The inset shows the presence of hollow core PANI nanofibers in the structure of the device.

With this method, 0.20 g (2.19 mmol) of ANI were added to 10.0 mL of 1.0 M HCl to prepare the monomer solution. An initiator solution was then prepared by adding 0.60 g (2.62 mmol) of APS to 10.0 mL of 1.0 M HCl and stirred for 5 min. Afterwards, the initiator solution was added dropwise to the monomer solution. The mixture was kept in an ice bath at a constant temperature of 0 °C and was magnetically stirred for 24 h to complete the polymerization procedure. During this process (Fig. 1a), the color of the mixture changed from an achromatic transparent solution of monomers to a pink pernigraniline solution. Then the solution became blue (leucoemeraldine), and finally changed to green due to the formation of the emeraldine-base form of PANI. The obtained mixture was poured into Falcon tubes, added together with the same volume of water, and centrifuged at 1900 rpm for 20 min. The water was then removed, and the same procedure was repeated using EtOH and then acetone, in order to remove any unwanted reaction residue.

0.2 g (1.73 mmol) of the synthesized emeraldine powder of PANI were then dissolved in 5.0 mL of DCE at 80 °C and stirred for 30 min. Subsequently, 0.1 mL of ChA was added to the obtained solution. After stirring for 15 min the obtained mixture was kept at 25 °C for 5 h. The resulting dark viscous precipitate was filtered through a membrane syringe filter and suspended in 10 mL of DI. The purified solution was then cooled down to 10 °C for 4 h. This step served to perform the polymer hydrolysis necessary to convert chlorosulfonic moieties into chlorosulfonic acid groups, in order to increase the final polymer solubility in water. After this step, the dark green precipitate that formed was separated from the solution by filtration, washed three times with acetone, and dried at 60 °C. The obtained polymer was used as a buffer layer in photovoltaic cells and its properties were compared with those resulting when PANI nanofibers are used.

FT-IR spectra of the synthesized products were collected on a BOMEM MB 100 spectrometer in the range of 4000 to 400 cm^−1^ with a resolution of 2 cm^−1^, and UV–Vis spectra were obtained by using an Analytik Jena Specord 210 operating between 200 and 800 nm.

### PAN electrospinning

Polyacrylonitrile (PAN) nanofibers were used as a sacrificial template for the preparation of PANI hollow nanofibers. A solution of 12% w/w of PAN in DMF was introduced into a 10.0 mL syringe – with a blunt-end metal needle and an inner diameter of 0.6 mm – and inserted into a syringe pump (Nano-fanavaran, 0–150 mL/h). A plane collector was set at a distance of 10 cm from the nozzle, and by connecting the positive pole of the voltage source to the needle tip and the negative pole to the collector, a voltage of 11.5 kV was applied while the feeding rate was set at 0.5 mL/h. Electrospinning was performed at room temperature and with a relative humidity of around 30%.

### Preparation of PANI@PAN nanofibers

The sacrificial PAN nanofiber was first immersed in 20 mL of a 1.0 M HCl solution containing 0.20 g (2.19 mmol) ANI, exposing the nanofibers to the monomer for 2 h. Afterwards, the reaction vessel was set into an ice bath to reach the necessary reaction temperature (T = 0 °C). A solution of 0.60 g (2.62 mmol) APS in 20.0 mL of 1.0 M HCl was then added dropwise into the reaction vessel to trigger the PANI polymerization reaction on the PAN nanofibers. After 24 h, 10.0 mL of acetone were added to the reaction vessel to complete the polymerization. The nanofibrous mat was then extracted from the solution, washed twice in acetone, and heated at 70 °C for 30 min. Lastly, the fibers were placed in a vacuum oven at 45 °C for 2 h to completely remove the remaining solvents.

### Preparation and characterization of hollow PANI fibers

Nanofibers were immersed in DMF for 24 h to selectively remove the PAN core from the fabricated core–shell fibers, while leaving the hollow PANI nanofibers unaltered. Subsequently, the hollow nanofibers were placed in a vacuum oven at 45 °C for 5 h to remove the remaining solvent.

The morphology of the electrospun nanofibers was characterized by using both a field emission scanning electron microscope (FE-SEM, Mira3, TESCAN) at an accelerating voltage of 10.0 kV and an atomic force microscope (AFM, EM3200, KYKY). The thermogravimetric analysis (TGA) was conducted under a nitrogen atmosphere using a Q5000 TGA (TA Instruments, USA) in the temperature range of 20–900 $^\circ$C, with a heating ramp of 10 $^\circ$C/min. ATR-FTIR spectra were recorded in the 4000–400 cm^−1^ range on a VERTEX 70 (Bruker) spectrophotometer to ensure that the PAN had been removed to achieve the desired hollow core PANI nanofibers. A 4-point probe method was used to investigate the conductivity of the samples at room temperature. For the cyclic voltammetry analysis, ITO was used as the working electrode, Ag/AgCl as the reference electrode, and platinum as the counter electrode, as well as 0.1 M solution of lithium perchlorate (LiClO_4_) in aqueous acetonitrile (CH_3_CN) as the electrolyte. 10.0 mg of each sample was dissolved in 1.0 mL of dichloro-benzene solvent and spin-coated onto 10 × 5 mm ITO glasses. The surface resistance and electrical conductivity of the samples was measured by a 4-point probe method using a Sanat Nama Javan device.

### Solar cell fabrication

The ITO substrate surface was first washed in a gentle cleaner and water to remove contaminants, after which the substrates were immersed in acetone, EtOH, and isopropanol, and treated with ultrasounds for 10 min at each stage. After the cleaning procedure, the ITO substrates were dried using a flow of nitrogen.

10.0 mg chlorosulfonated PANI powder were mixed in 10 mL of DI for 4 h at 100 °C to perform hydrolysis and obtain a chlorosulfonic acid group. The aqueous PANI solution was sonicated for 40 min before layering; then it was filtered by a 0.45-µm syringe filter until a uniform green solution was obtained. To cover the ITO surface with the PANI, a spin coater with a disc rotating at 3000 rpm was used for 3 min. Lastly, the prepared buffer layer was put into a 50 °C oven for 5 min.

To create a uniform layer made of electrospun PANI nanofibers, a dispersion of 10.0 mg of PANI hollow fibers in 1.0 mL of NMP was deposited on the glass slide to cover the ITO layer, then a backfilling layer was created on the fiber layers by doctor blading^22^. After the layer fabrication, the sample was heated at 50 °C for 5 min to fix the structure completely.

To prepare the active layer solution, 0.1 g of P3HT was mixed with 0.1 g of PCBM in 1.0 mL of DCB. The resulting solution was spin-coated onto the buffer layer's surface with a rotation of 1500 rpm for 4 min. The sample was then placed on a hot plate at 70 °C for 10 min^28^.

The last step was aluminum electrode deposition on polymer layers. The prepared samples were placed in a vacuum chamber for aluminum coating at a 6^–10^ Pa pressure to obtain a 100 nm-thick layer. The thickness of each layer is as follows: ITO (100 nm), buffer layer (90 nm), P3HT:PCBM (150 nm), and Al as an electrode (100 nm).

## Results and discussion

We used the above-mentioned steps to polymerize emeraldine PANI, which was used as a buffer layer for the organic solar cell. A schematic illustration of the chemical reactions performed to synthesize PANI and sulfonated PANI has been shown in Fig. 1a. We used the electrospinning and sacrificial template method to improve the obtained emeraldine PANI performance for solar cells. These steps led us to introduce hollow core PANI nanofibers as a novel buffer layer in organic solar cells. Figure 1b shows a schematic view of the techniques used to achieve these nanofibers, while Fig. 1c is a sketch showing the architecture of the developed organic solar cell and the presence of hollow core nanofibers in the buffer layer.

**Figure 2:**
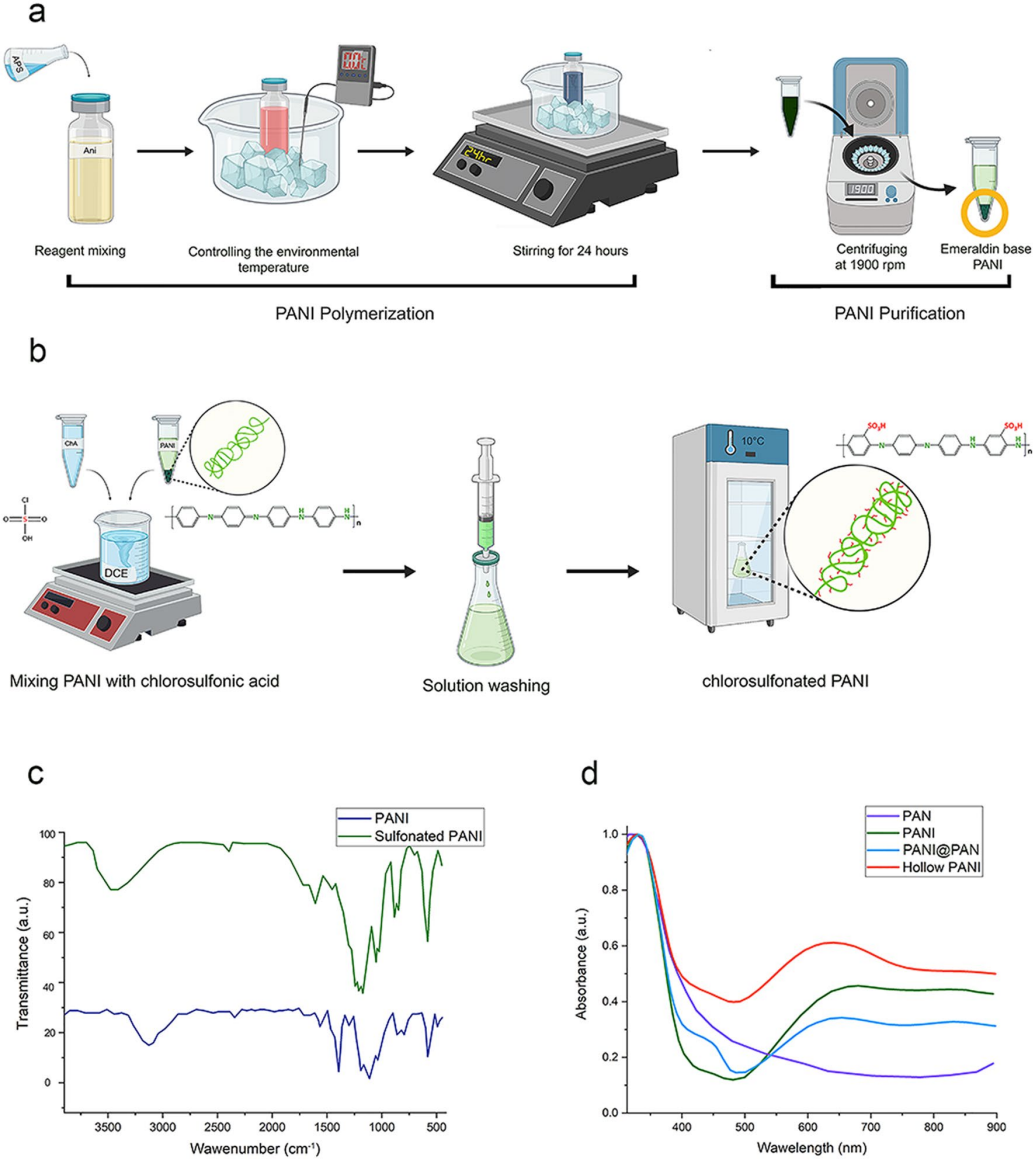
Step-by-step development of hollow core nanostructured fibers. (**a**) Sketch showing the synthesis of emeraldine base PANI starting from monomers. (**b**) Chemical reaction scheme to obtain sulfonated PANI. (**c**) FT-IR spectra of PANI and sulfonated PANI showing the absorption of the sulfonic group at around 3450 cm^−1^. (**d**) UV–Vis absorption spectra of the four polymer structures: PAN, PANI, PANI@PAN, and hollow core PANI nanofibers.

The schematic synthesis and purification of PANI is shown in Fig. 2a, while a graphic representation of the PANI chain functionalization using a sulfonating agent to increase the hydrophilicity of polymer is shown in Fig. 2b.

Figure 2c shows the FTIR spectra of PANI and sulfonated PANI collected in the range between 400 and 4000 cm^−1^. PANI showed peaks at 1583 cm^−1^ and 1483 cm^−1^, which can be ascribed to the stretching of C–C and C–N in the quinoid and benzenoid rings, respectively, thus confirming the formation of amine and imine units in the polymer backbone. Moreover, the intensity of quinoid and benzenoid peaks reveals the oxidation state of PANI. Characteristic peaks of C–N stretching of the benzenoid unit are visible at 1296 cm^−1^, proving PANI protonation. Moreover, two peaks appeared at 811 and 1114 cm^−1^, corresponding to the out-of-plane bending and in-plane bending vibration of C-H in the aromatic ring and quinoid structure, respectively. Weak bands at 3438 cm^−1^ and 698 cm^−1^ are ascribable to the –OH bonds of SO_3_H groups in the ortho position obtained after sulfonation. C–S stretching vibration peaks are visible in the range of 600–700 cm^−1^, indicating the existence of SO_3_H functional groups on the aromatic rings, which are less significant due to the low intensity and displacement in molecular structure^29^.

Figure S1 shows the FT-IR spectra of ANI and PANI. In the PANI spectrum, peaks at 1568 cm^−1^ indicate the vibrations of the C=N bond, which confirm the effectiveness of the PANI polymerization. The C-N related peak observed at around 1275 cm^−1^ confirms the protonation of the synthesized PANI. Furthermore, the characteristic peaks observed at around 700 and 3480 cm^−1^ are attributed to the N–H bond vibrations in the aniline ring, which can be seen in both samples. The presence of the above-mentioned peaks is an evidence of the synthesis of PANI in the protonated state of emeraldine.

In general, an efficient buffer layer for organic solar cells requires sufficient conductivity to reduce resistance between layers. To evaluate the effect of nanofibers on the conductivity of the polymer, a four-point probe method was used. Surface resistance values of emeraldine PANI, sulfonated PANI and hollow core PANI are shown in Table S1. According to these results, functionalization of PANI to increase hydrophilicity leads to an increased surface resistance of the polymer and a decreased conductivity compared to the emeraldine PANI. As predicted, the hollow core PANI nanofibers led to an increased conductivity of the polymer. Figure S2 shows a comparison between the conductivities of different types of PANI used in this research.

UV–Vis spectroscopy is a simple and effective method for monitoring absorbance and band gap of materials. The UV–Vis spectra of PANI, PAN NFs, hollow PANI NFs, and PANI NFs are shown in Fig. 2d. The UV–Vis spectroscopy of the mentioned polymers was performed by analyzing thin films of polymers on quartz slides. Spectra are characterized by the presence of two main absorption bands in the 300–340 nm and in 527- 590 nm ranges ascribable to π–π* transition in the benzenoid rings related to the exciton absorption of quinoid rings; benzenoid ring peaks shifted to a higher wavelength, while the quinoid ring peak shifted to a lower wavelength in hollow PANI. The absorption of the buffer layer reduces the total energy which can be produced by the solar cell. In fact, the use of HTL is a delicate balance between the enhancement of charge mobility and the loss of light energy that can be absorbed by the active layer.

**Figure 3:**
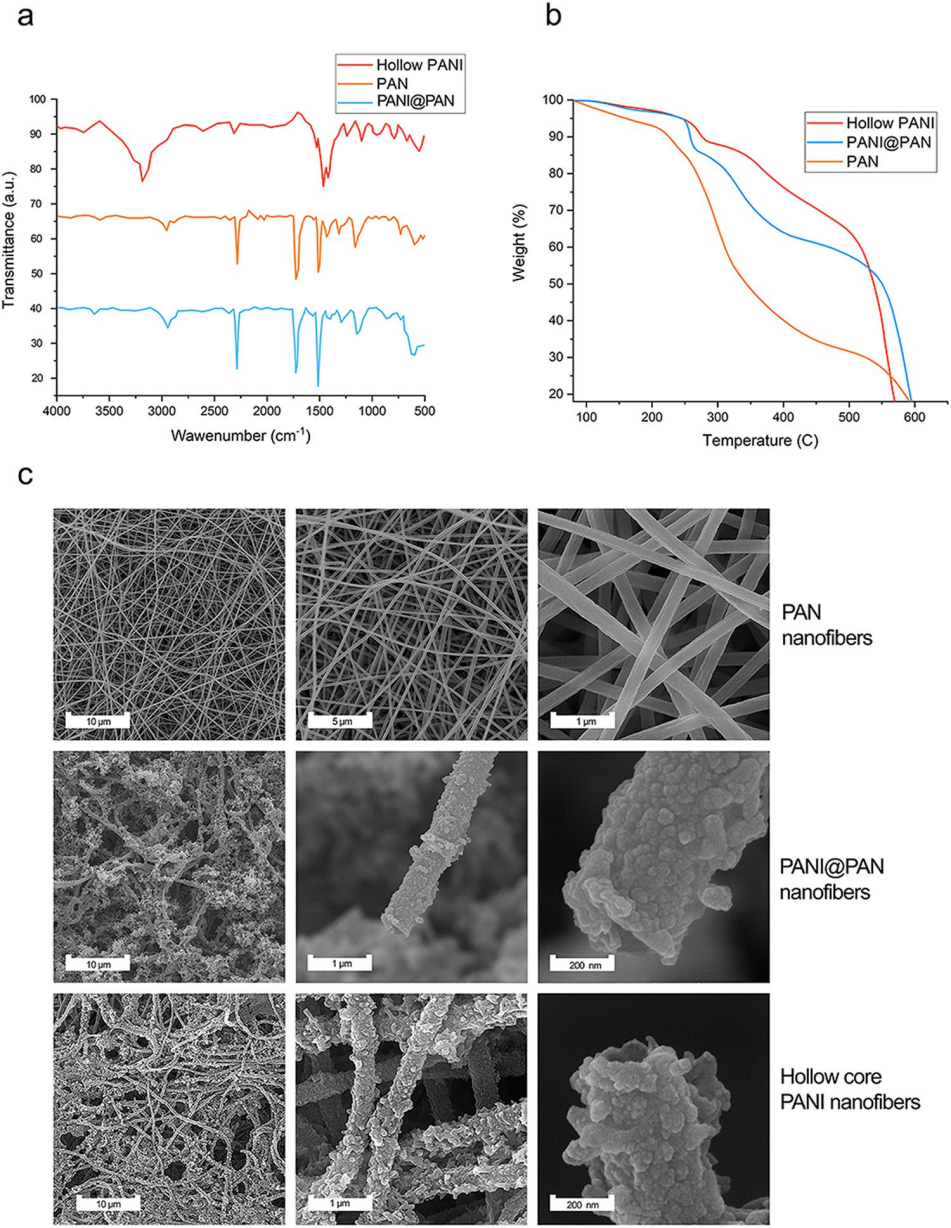
Morphological, structural, and thermal features of the fabricated electrospun nanofibers. (**a**) ATR-FTIR spectra of hollow PANI, PAN, and PANI@PAN. Lack of an absorption peak at 2243 cm^−1^ in the hollow PANI sample implies that there is no PAN on the surface of the hollow nanofibers; (**b**) TGA gravimeters to investigate the PAN removal procedure as a sacrificial template; (**c**) FE-SEM tests conducted on nanofibers of PAN, PANI@PAN and hollow core PANI. The cross-sections of the hollow nanofibers clearly show their hollowness.

The characteristic peak of cyanide groups appearing in the ATR-FTIR spectrum of PAN nanofibers around 2250 cm^−1^ (Fig. 3a). The transmission peaks of PANI@PAN nanofibers are around 1500 and 1459 cm^−1^ attributed to C–C stretching of quinoid and benzenoid groups. C–N stretching of aromatic band of amine and C-H bending vibration transmission peaks were shown at wavelengths around 1150, 1300 and 800 cm^−1^, respectively for PANI nanofibers. Attendance of the characteristic peaks of both quinoid and benzenoid groups indicates that the synthesized polymer is in the chemical structure of emerald salt. Moreover, the peak that appeared at 2200 cm^−1^ is due to the cyanide groups of PAN nanofibers. The spectrum of the hollow core PANI nanofiber shows similar peaks to that of the core–shell PANI@PAN nanofibers, except that the peaks attributed to the cyanide group are not present. The imperceptible peak of the cyanide group in the hollow core nanofibers implies that the sacrificial template method was performed^30^.

The TGA thermograms in Fig. 3b show the removal of the PAN core in the nanofibers. Two prominent weight loss peaks at about 260 °C and 570 °C are observed in the TGA curves. The first polymer weight loss can be ascribed to the removal of the carboxyl group and the release of carbon dioxide of the methyl acrylate in PAN nanofibers, while the second weight loss was deterioration due to the oxidation of the PAN polymer chains. The initial value of the first weight loss rose when PANI was present in the structures, while the temperature of the second weight loss decreased with increasing PANI content. This is the reason that most of the PAN core of the nanofibers could be removed, resulting in the hollow nanofibers of PANI^31^. Anyway, it is worth mentioning that TGA curves evidenced that even if the large majority of PANI@PAN nanofiber core is removed during the post-electrospinning process, a small amount of PAN (around 6.5%) remained trapped into the core of the hollow nanostructures.

FE-SEM images at a different magnification of PAN, core–shell PANI@PAN, and hollow core PANI nanofibers are shown in Fig. 3c. As can be seen, PAN nanofibers have no beads and are geometrically uniform, with an average diameter of 250 ± 25 nm. PANI@PAN nanofibers show uniform core/shell fibers characterized by the typical textured surface and a mean diameter of 450 ± 32 nm. According to Fig. 3c, hollow core PANI nanofibers with an inner diameter of 350 ± 25 nm have a unique tube-shaped morphology, confirming the complete removal of the PAN core.

**Figure 4:**
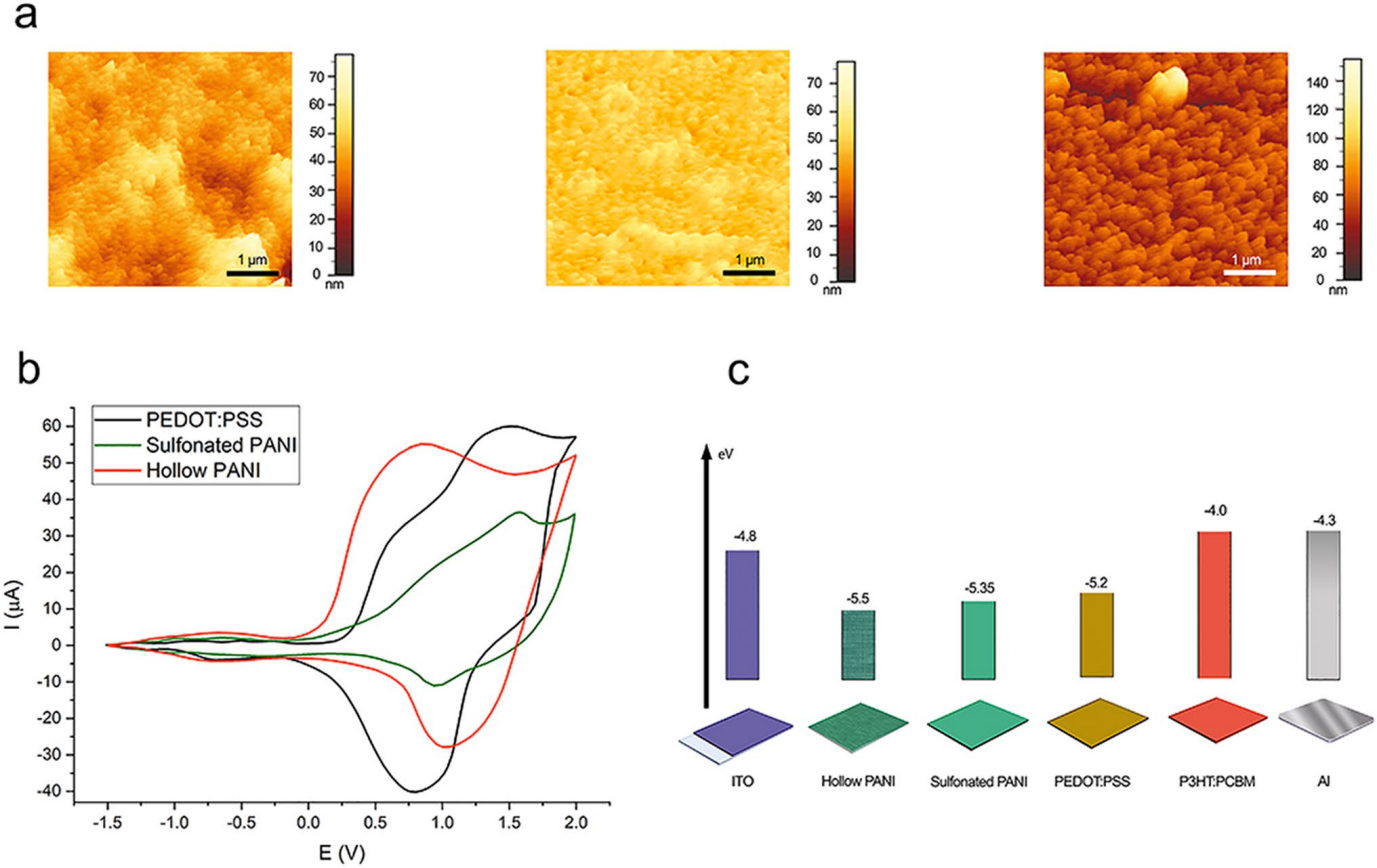
Structural and electrochemical properties of the developed organic solar cell. (**a**) AFM topographical maps of the buffer layer surface structure made of sulfonated PANI, PEDOT:PSS, and hollow core PANI. The AFM image z-scales are 77 nm (PANI), 78 nm (PEDOT:PSS), and 154 nm (hollow core PANI). (**b**) Investigation by cyclic voltammetry of sulfonated PANI, PEDOT:PSS, and hollow core PANI to estimate the optical properties of buffer layers, and (**c**) schematic of the energy levels for all the layers used in solar cells.

Atomic force microscopy (AFM) was performed to analyze the roughness and morphology of the surface of the synthesized buffer layers. Figure 4a shows the topography results of three PEDOT:PSS, sulfonated PANI, and hollow core PANI nanofibers. As shown, the hollow core nanofibers coated on the ITO surface have lower roughness than the PEDOT: PSS and sulfonated PANI samples. This roughness can improve the solar cell performance by affecting the hole transfer. The root- mean- square (rms) roughness of the hollow core PANI was 31.04 nm, while in the case of sulfonated PANI was 42.08 nm. For comparison purposes and to a better understanding of the roughness changes in buffer layers, we also analyzed PEDOT:PSS layers, which had an rms of 72.06 nm.

Figure S3 shows Tauc curves for the calculation of PANI and hollow core PANI nanofiber energy gaps (E_g_) using the results of the UV–Vis analysis. The E_g_ values calculated for PANI and hollow PANI are shown in Table S2. According to these values, hollow nanofibers reduce the band gap in PANI.

Cyclic voltammetry was performed to estimate HOMO and LUMO levels in the polymers used in order to compare their energy band gap values. Three samples were subjected to this analysis. The values of E_ox_ and E_red_ are determined from voltammograms and set into Eqs. (1)–(3)^32^.

1 $${\text{E }}\left( {{\text{HOMO}}} \right) \, = \, - {\text{e}}\left[ {{\text{E}}_{{{\text{ox}}}}^{{{\text{onset}}}} + { 4}.{4}} \right]$$

2 $${\text{E }}\left( {{\text{LUMO}}} \right) \, = \, - {\text{e}}\left[ {{\text{E}}_{{{\text{red}}}}^{{{\text{onset}}}} + { 4}.{4}} \right]$$

3 $${\text{E}}\,\left( {{\text{LUMO}}} \right){-}{\text{E}}\,\left( {{\text{HOMO}}} \right) = {\text{E}}_{{\text{g}}}$$

Figure 4b shows the voltammetric curve of PEDOT:PSS. From this curve, an oxidation potential of 0.81 eV and a reduction potential of −1.50 eV can be calculated and, according to the above-mentioned formulas, the values estimated for the HOMO and LUMO levels are −5.20 and −2.89 eV, respectively. Therefore, the band gap level of this sample is 2.31 eV. Figure 4b shows the results of the analysis on sulfonated PANI. The reduction potential is −1.59 eV, while the oxidation potential is 0.95 eV, producing LUMO and HOMO levels of −2.81 and −5.35 eV, respectively, and a band gap of 2.54 eV. As a result, the band gap is greatly increased, which reduces the solar cell efficiency. The cyclic voltammetry analysis conducted on hollow core PANI nanofibers showed that the reduction potential was −0.87 eV, while the oxidation potential was 1.10 eV. Similarly, HOMO (−5.50 eV) and LUMO (−3.53 eV) levels and the energy band (1.97 eV) were also calculated. The obtained results indicate that as the energy band decreases, the efficiency of the polymer solar cell increases.

Table 1 shows the comparison of HOMO, LUMO, E_ox_, E_red_, and E_g_ levels for different samples, as shown in Fig. 4c. As can be seen, sulfonated PANI has the lowest E_red_ and the highest E_g_, meaning that it does not perform ideally as a buffer layer. On the other hand, hollow core nanofibers have a lower band gap, even lower than that of PEDOT:PSS. This decrease in band gap results in a higher conductivity and better hole transport when the material is used as a buffer layer for solar cells.

**Table 1:** Energy levels of PEDOT:PSS, sulfonated PANI and hollow core nanofibers calculated by CV analysis.

	E_ox_ (eV)	HOMO (eV)	E_red_ (eV)	LUMO (eV)	E_g_ (eV)
PEDOT:PSS	0.8	−5.2	−1.51	−2.89	2.31
Sulfonated PANI	0.95	−5.35	−1.59	−2.81	2.54
Hollow PANI	1.10	−5.50	−0.87	−3.53	1.97

**Figure 5:**
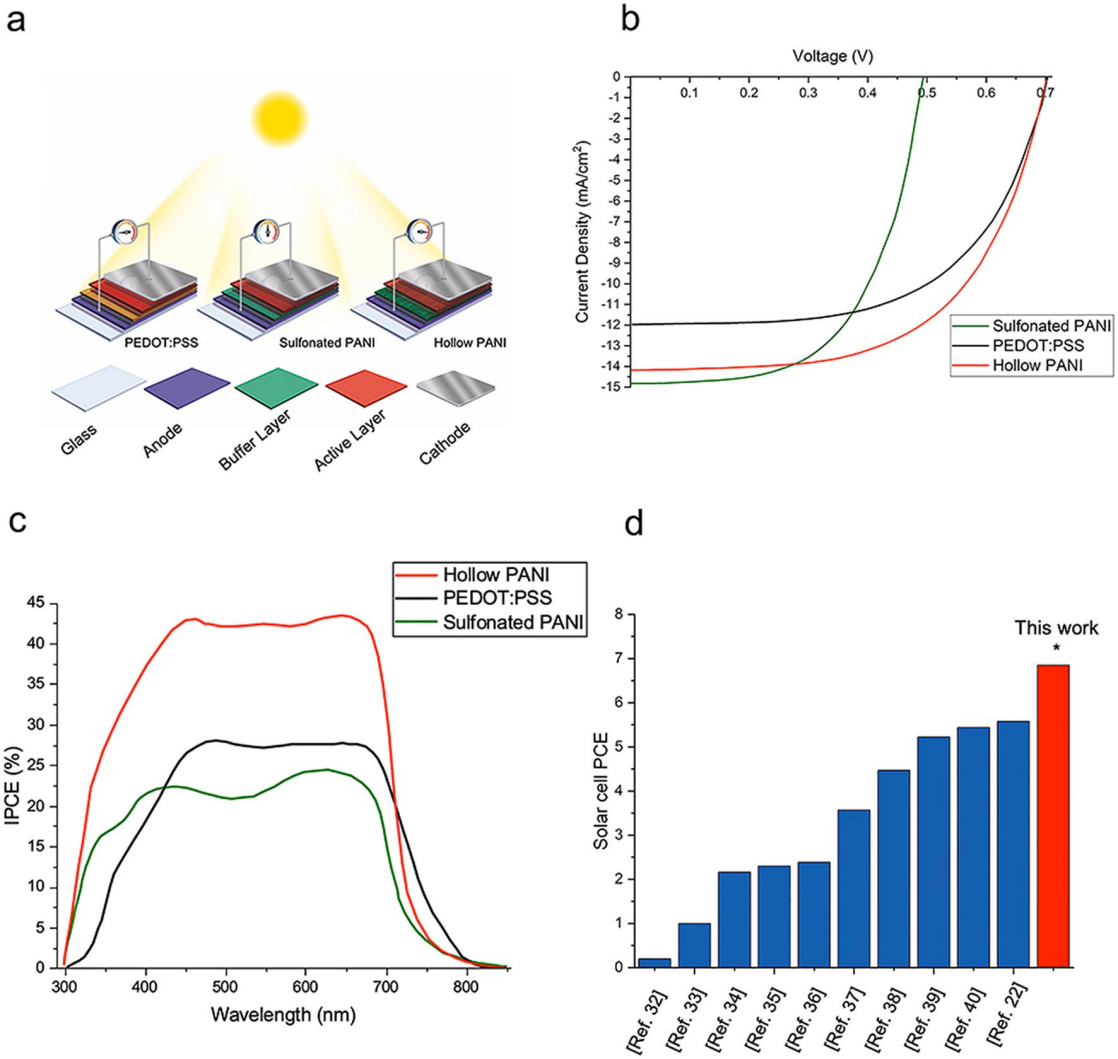
Photovoltaic performance of polymer solar cells. (**a**) Schematic of the manufactured solar cells with each of the constituent layers. (**b**) Performance of synthesized buffer layers via J-V characterization of different polymer solar cells under simulated AM1.5G sunlight illumination. (**c**) IPCE spectra of the polymer solar cells fabricated using PANI, PEDOT:PSS and hollow core PANI as buffer layers. (**d**) Comparison of the efficiency of this research with previous studies in the field of organic solar cells over the past ten years.

A schematic of the solar cells and a comparison of their performances is shown in Fig. 5a. Hollow core nanofibers can improve the efficiency in several ways: by increasing the electrical conductivity of the buffer layer, shortening the hole pathway to improve hole extraction, decreasing the surface roughness of the buffer layers, and improving the exciton generation^11^.

Tests performed with a solar simulator to calculate important solar cell factors such as power conversion efficiency (PCE), open circuit voltage (Voc), short circuit current (Jsc) and fill factor (FF). Standard air mass 1.5 and 1000 W/m^2^ conditions were applied to obtain this analysis^33^. The photovoltaic parameters of the measured devices are shown in Fig. 5b and Table S3. The reference solar cell with a typical buffer layer made of PEDOT:PSS, shows Voc of 0.70 V, Jsc of 11.97 mA/cm^2^ and FF of 0.52. The calculated PCE is thus 4.35%. BHJ solar cell of sulfonated PANI shows the Voc of 0.49 V, Jsc of 14.82 mA/cm^2^ and FF of 0.50. Therefore, the calculated PCE was 3.63%. This decrease in PCE is due to the lower conductivity of sulfonated PANI compared to PEDOT:PSS.

The visible difference in current density value is related to the morphology of the photoactive layer, which can also affect the charge transport. The highest value of Jsc was obtained with the hole transport layer of sulfonated PANI, which showed the lowest electrical conductivity. However, the ionic conductivity in sulfonated PANI solar cells, related to the flow of sulfur trioxide and phenyl ammonium ions (and not to the flow of electrons) can explain the higher current density value obtained with this buffer layer, also considering that the aforementioned ions can generate charge transfer complexes with P3HT with positive effects on the photovoltaic parameters. After deployment of the PANI hollow core buffer layer, Voc remained almost unchanged, while Jsc reached 14.18 mA/cm^2^ and PCE increased up to 6.85%. The fill factor of the solar cells made of hollow core PANI nanofibers is 27% higher than that of PEDOT:PSS. The reason for this increase in performance may be related to the increase in the level of optoelectronic properties of the layer, the decrease in its surface roughness and the increase in conductivity.

The results of IPCE analysis are shown in Fig. 5c to quantify the enhancement obtained in terms of Jsc and PCE. As expected, photon-to-current conversion was enhanced by the use of PANI hollow core nanofibers. A distinguishable enhancement in the IPCE around the visible spectrum, which is equal to sunlight wavelength, assigned to the improvement in optical properties, can be observed in the hollow core PANI device.

Lastly, we compared the effectiveness of this research with previous works. In the field of organic solar cells with a nanofiber structure, we introduced hollow core nanofibers as a novel and effective buffer layer of organic solar cells. In this research, we developed a high-efficiency organic solar cell made of electrospun nanofibers, which represents an important step forward in this field, as is apparent from the comparison of our results with those reported in the past ten years of studies^7,22,34–42^. The graph in Fig. 5d shows the efficiency achieved in this study in comparison with the levels obtained in previous ones.

## Conclusions

In summary, well-defined polymer solar cells incorporating a hollow core PANI structure were successfully fabricated and characterized. The new morphology and nano-structuration of hollow core PANI nanofibers showed a better conductivity compared to PANI. Moreover, the presence of the novel hierarchical hollow core structure improved the transport of charge carriers, while enhancing the efficiency of polymer solar cells. Structural and chemical characterizations clearly show the formation of hollow core polymer nanofibers by electrospinning. Experimental results have shown the improved conductivity properties of hollow core PANI (conductivity increased by around 58%). Solar cell photovoltaic parameters showed higher short circuit current (around + 20%) and power conversion efficiency (around + 48%) than the reference, when the nanostructured interlayer is incorporated. As these results indicate, the hollow core structure of PANI can efficiently be used to produce future high-performance PSCs thanks to its straightforward procedure of synthesis, low cost, high absorbance, and effective charge transfer. These results demonstrated that hollow core conductive polymers could become an alternative rational design in the buffer layer of photovoltaic devices.

## Supplementary Information

Supplementary Information.
