# Supplementary material for: Enhanced efficiency in hollow core electrospun nanofiber-based organic solar cells
Source: Sci Rep. 2021 Oct 27;11:21144. doi: 10.1038/s41598-021-00580-4 (PMC8551186; doi:10.1038/s41598-021-00580-4)
Supplement: Supplementary file 1 — Supplementary Information. [file 41598_2021_580_MOESM1_ESM.docx]

Supplementary Information for

Enhanced Efficiency in Hollow Core Electrospun Nanofiber-Based Organic Solar Cells

Mohammad Ali Haghighat Bayan ^1,2^, Faramarz Afshar Taromi ^2^, Massimiliano Lanzi ^3^ and Filippo Pierini ^*1^

1. Department of Biosystems and Soft Matter, Institute of Fundamental Technological Research, Polish Academy of Sciences, Warsaw 02-106, Poland

2. Department of Polymer Engineering, Amirkabir University of Technology, Tehran 15875-4414, Iran

3. Department of Industrial Chemistry“Toso Montanari”, Alma Mater Studiorum – University of Bologna, Bologna 40136, Italy

**Corresponding Author:** *Email:fpierini@ippt.pan.pl

**Supplementary Figures and Tables**


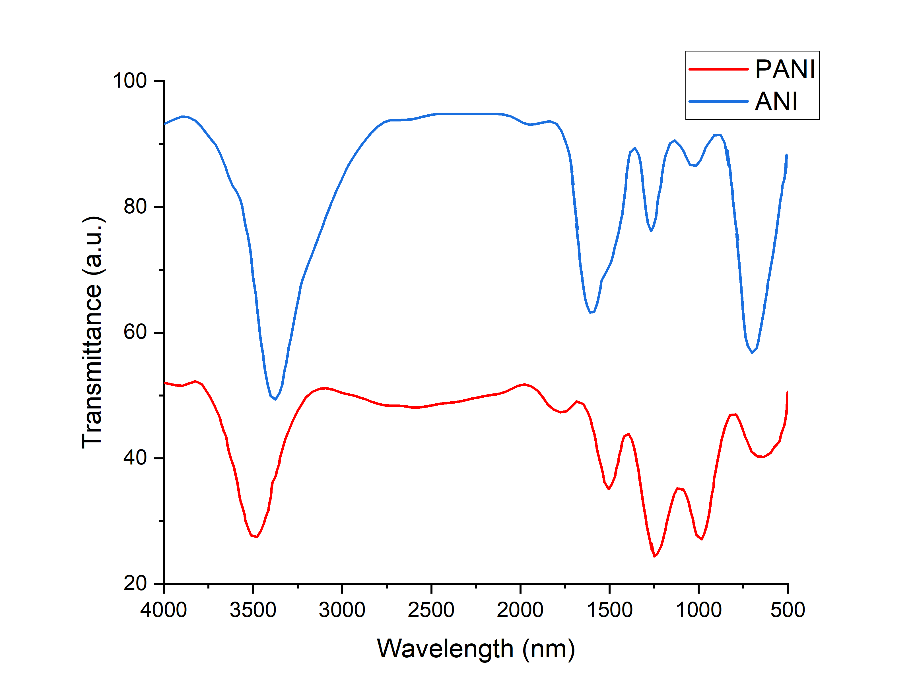


**Figure S1.** FT-IR spectra of PANI and ANI to show the polymerization.

**Table S1.** Surface resistance and conductivity of Sulfonated PANI, PANI, and Hollow PANI

| Sample | Conductivity (S/cm) | Resistance (Ohm/sq) |
| --- | --- | --- |
| Sulfonated PANI | 19.70 ± 3.45 | 2.05*10^4^±359 |
| PANI | 24.35 ± 1.39 | 1.41*10^4^±80.48 |
| Hollow PANI | 47.95 ± 0.79 | 5.84*10^3^±96.21 |


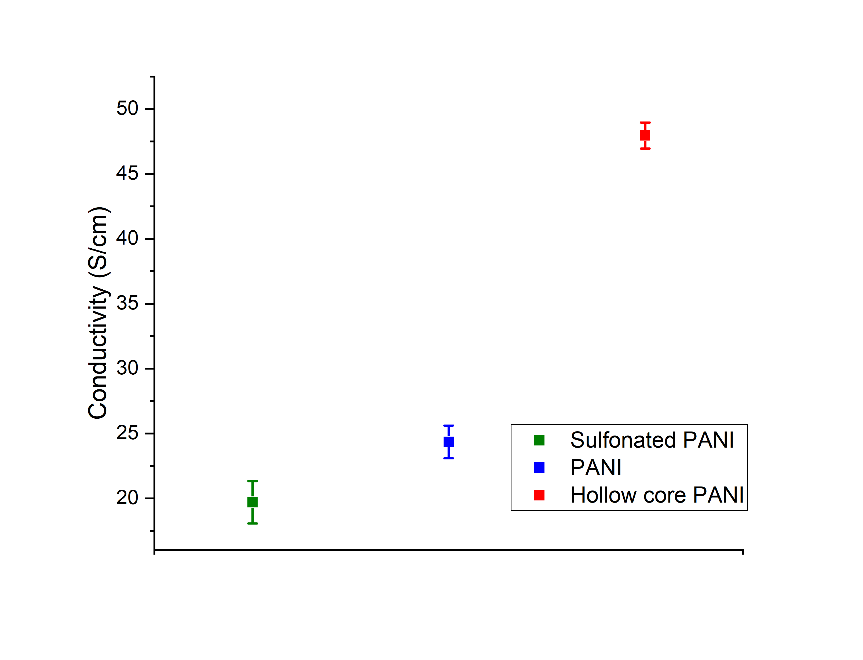


**Figure S2.** Change in conductivity of samples.


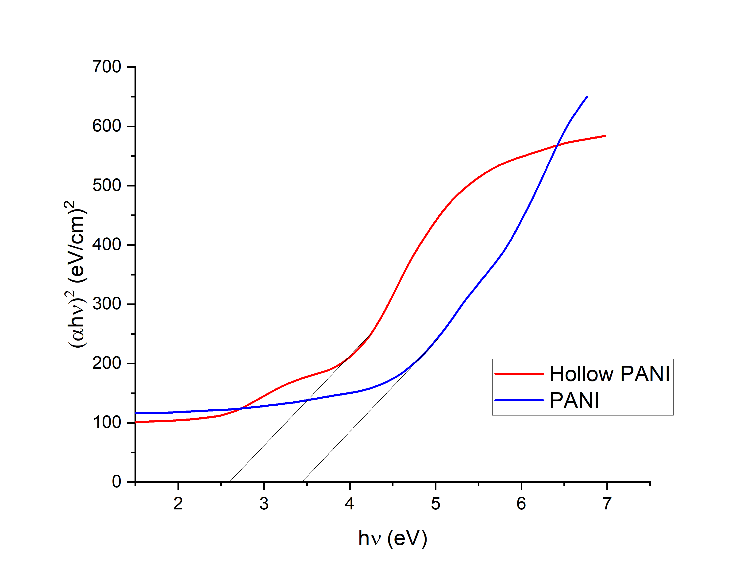


**Figure S3.** Tauc plot, derivated from UV-Vis data to estimate the optical Eg of PANI and Hollow PANI.

**Table S2.** Optical ban gaps for PANI and Hollow PANI.

| Sample | Band gap (eV) | Maximum absorption (nm) |
| --- | --- | --- |
| PANI | 3.45 | 260 |
| Hollow PANI | 2.60 | 219 |

**Table S3.** Photovoltaic characteristics of the examined solar cells (average values collected from five devices)

| Sample | J_sc_ (mA cm^-2^) | V_oc_ (V) | FF | PCE (%) |
| --- | --- | --- | --- | --- |
| PEDOT:PSS | 11.97±1.2 | 0.70±0.01 | 0.52±0.05 | 4.35±0.08 |
| Sulfonated PANI | 14.82±1.4 | 0.49±0.01 | 0.50±0.07 | 3.63±0.07 |
| Hollow PANI | 14.18±1.3 | 0.71±0.01 | 0.68±0.06 | 6.85±0.11 |
